# Comparison and Three-Dimensional Fluorescence Spectrum Analysis of Activated Sludge Treatment with Fenton and UV-Fenton

**DOI:** 10.3390/microorganisms11123003

**Published:** 2023-12-18

**Authors:** Jiamei Wang, Tian Chai, Xin Chen

**Affiliations:** College of Environmental Science & Engineering, Xiamen University of Technology, Xiamen 361024, China; wangjiamei1220@163.com (J.W.); 2022031238@s.xmut.edu.cn (X.C.)

**Keywords:** activated sludge, Fenton, UV-Fenton, crack, 3D-EEM, SEM

## Abstract

This study investigated the effects of single Fenton and Fenton and UV combined processes on the cracking degree of anaerobic sludge under the same conditions. The optimal experimental conditions were obtained by repeated determination of Fe^2+^ dosage, H_2_O_2_ dosage and reaction time, so as to achieve the maximum cracking of sludge. In addition, this study applied three-dimensional fluorescence spectrum analysis technology to analyze the organic matter leached from the treated sludge, and different regions of the three-dimensional fluorescence spectra were analyzed and compared for each treatment condition. Repeated experiments showed that the optimal conditions for Fenton are a pH of 3, reaction time of 40 min, 1.4 g/L of Fe^2+^ and 9 g/L of H_2_O_2_. The Fenton process cracking yielded a protein concentration of 0.66 mg/L and sCOD of 5489 mg/L, and the UV-Fenton pretreatment yielded a protein concentration of 0.74 mg/L and sCOD of 5856 mg/L. The sludge particle size was reduced from the original 54.52 mm to 40.30 mm and 36.37 mm, respectively. In addition to these parameters, it was also demonstrated that the Fenton process has a strong cracking effect on sludge by indicators such as the SEM and sludge water content and that UV irradiation can play a role in assisting and helping sludge cracking.

## 1. Introduction

At present, the most effective and mature technology for treating sewage in China is the activated sludge method, which is more effective in treating industrial wastewater and domestic sewage, and at the same time, its construction investment is relatively low. However, the activated sludge method of sewage treatment also has a big disadvantage, that is, a large amount of sludge will be produced in the process of sewage treatment in sewage plants. The problem of sludge treatment has become an important issue in the sewage treatment process [1]. The main components of sludge are water, microorganisms and extracellular polymers (EPS) [2], and sludge is a multiphase colloidal system with a complex composition, containing a large number of bacteria and environmentally harmful pollutants, such as dioxins and heavy metals [3]. At the same time, sludge cells are also rich in organic matter, so for more effective resource utilization, the floc structure of sludge needs to be cracked to extract the organic matter in the sludge cells and in the EPS, so that the organic compounds in the cells can be more easily utilized [4]. In order to use the organic matter in sludge more efficiently, it is necessary to pretreat the sludge, crack the complex floc structure of sludge by physical, chemical and biological methods, break the sludge cells, release the intracellular organic matter [5] and eliminate the hydrolysis resistance of certain organic macromolecules [6]. Pretreated sludge is more conducive to being used as a nutrient substrate for the processes of anaerobic fermentation gas and acid production, in accelerating the fermentation process in the hydrolysis stage and in achieving resourceful utilization of the sludge while improving its utilization efficiency. Zhang, et al. [7] showed that different pretreatments of sludge can potentially reduce the hazards caused by bacteria and pathogens while mitigating the contamination caused by heavy metals. Wang et al. [8] used a new mechanically cut pretreatment (MCP) method for sludge pretreatment and the pretreatment sCOD could reach 1206 mg/L, which promoted the hydrolysis and acidification process of sludge fermentation and anaerobic digestion. Qiao et al. [9] used the strategy of pretreatment of sludge by oxidation with potassium pertechnetate (K_2_FeO_4_), which mainly achieved the promotion of sludge hydrolysis and the degradation of some antibiotics in the sludge.

Advanced oxidation processes are efficient in treating sewage and sludge, with a strong oxidation capacity, zero secondary pollution and other characteristics [10]. As an advanced oxidation process, the Fenton process has attracted more and more attention because of its advantages of being friendly to the environment and minimizing excess sludge [11]. The Fenton reaction needs to be carried out under strongly acidic conditions. It decomposes H_2_O_2_ under the catalytic effect of Fe^2+^ and produces a large number of strong oxidizing intermediate hydroxyl radicals (·OH) [12] without toxic byproducts.

The conventional Fenton process involves a free radical chain reaction [13]:

$$\begin{array}{l} Fe^{2+}+H_{2}O_{2}\to Fe^{3+}+OH^{-}+\cdot OH\\ Fe^{2+}+\cdot OH\to Fe^{3+}+OH^{-}\\ Fe^{3+}+H_{2}O_{2}\to Fe^{2+}+\cdot HO_{2}+H^{+}\\ Fe^{3+}+\cdot HO_{2}\to Fe^{2+}+O_{2}+H^{+} \end{array}$$

The mechanism of the Fenton process is well understood by researchers, who have found that oxidation is the main effect on sludge. Oxidation is generated by the ·OH in the above reaction, and its REDOX potential is extremely high, up to 2.8 V, which can quickly oxidize and crack EPS, thus achieving the purpose of cracking the sludge and changing the sludge’s structure.

The Fenton process has its shortcomings, such as poor utilization of H_2_O_2_ and an inability to achieve very low acidic conditions under certain circumstances. Therefore, it is necessary to improve the Fenton process by using ultraviolet irradiation to cooperatively catalyze the reaction process and promote the generation of a large amount of ·OH [14]. The purposes of this study were to study the Fenton process and the ultraviolet Fenton (UV-Fenton) process, carry out a comparison of four experimental conditions, such as the same acidity required by the reaction and ultraviolet irradiation, and explore the research through the detection of soluble chemical oxygen demand (sCOD), total organic carbon (TOC), protein, moisture content and other indicators. The extracellular polymers (EPS) of sludge were characterized by a three-dimensional fluorescence spectrometer to intuitively determine the composition of the effluent. Three-dimensional fluorescence spectroscopy (3D-EEM) is a rapid and highly sensitive technique that is often used to characterize dissolved organic matter in water and soil [15]. The advantage of 3D-EEM is that it does not damage the sample, has good selectivity and can analyze the protein and humic acid in the natural environment. Therefore, it can be used to study the EPS of sludge [16]. The effect of sludge particle size on the degree of and reduction in sludge cracking was observed using laser particle sizing. In addition, the morphology of the sludge before and after cracking was characterized by scanning electron microscopy (SEM).

## 2. Materials and Methods

### 2.1. Chemicals and Machinery

Hydrogen peroxide (H_2_O_2_), ferrous sulfate heptahydrate (FeSO_4_·7H_2_O), sulfuric acid (H_2_SO_4_), sodium hydroxide (NaOH) and anaerobic sludge medium (anhydrous sodium acetate, ammonium chloride, disodium hydrogen phosphate, anhydrous calcium chloride, magnesium sulfate heptahydrate and potassium dihydrogen phosphate) were obtained.

A high-speed centrifuge, multi-parameter tester (5B-6C(V10)), visible spectrophotometer and TOC analyzer (Sievers InnovOx ES Lab, Boulder, CO, USA) were used.

### 2.2. Activated Sludge

The activated sludge used in this study was taken from a sewage treatment plant in Jimei District, Xiamen City, Fujian Province, which treats 6 × 10^4^ m^3^ of sewage per day. Its main process is A_2_/O treatment. The activated sludge samples were directly transported to the laboratory for culture treatment after retrieval. The basic indexes of the raw sludge samples were determined. The initial pH of the samples was 7.2 ± 0.15, the sCOD was 870 mg/L, the TOC was 158 mg/L, the protein content was 1.42 × 10^−3^ mg/L, the polysaccharide content was 2.3 × 10^−2^ mg/L and the humic acid content was 3.3 × 10^−4^ mg/L.

### 2.3. Fenton and UV-Fenton Processes

The experimental conditions of this study were the optimal reaction conditions obtained after many experiments. Different concentrations of FeSO_4_ and a dosage of 30% H_2_O_2_ were used as reaction reagents to carry out the Fenton experiments. Repeated experiments have proven that the best pH is between 2 and 4 [16]. This experiment was carried out under the condition of a pH of 3 in an acidic environment. A total of 1 mol/L of H_2_SO_4_ was used to adjust the pH of the sludge to 3, and then Fe^2+^ of different concentrations was added successively. The sludge mixture was fully stirred for 15 min, and then H_2_O_2_ was added for 40 min of reaction. When the reaction time was reached, the reaction was terminated by adjusting the pH of the reaction system to neutral. The reaction time was timed from the addition of H_2_O_2_. The entire Fenton reaction was performed using a magnetic stirrer at 250 rpm. At the end of the reaction, the sludge mixture was centrifuged at 8000 r/min for 10 min to separate the solid–liquid to extract the organic matter, and finally the indicators were determined.

**Figure 1:**
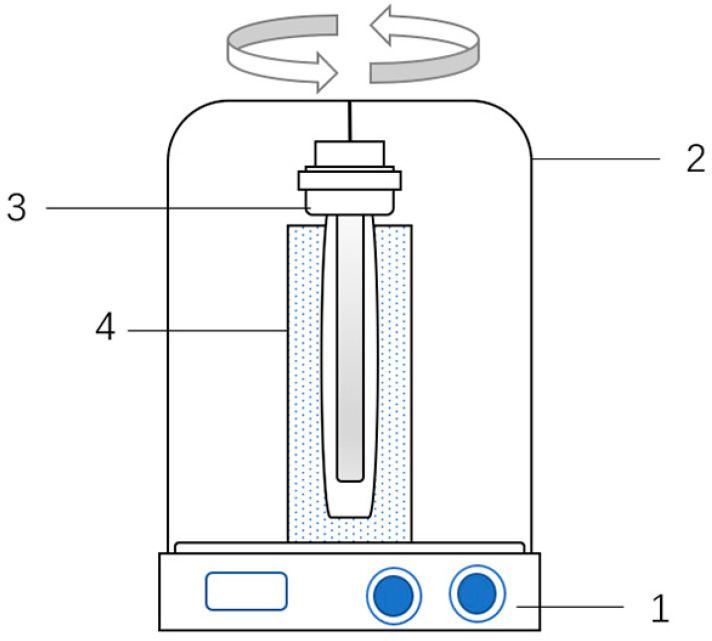
UV-Fenton reaction device. 1—Magnetic stirrer; 2—Light shield; 3—UV lamp; 4—Test sludge and light repellent bottle.

On the basis of the Fenton process, a UV lamp with a wavelength of 254 nm was used to assist in the experiment. As shown in Figure 1, the experiment was conducted under fully closed and shaded conditions, and the sludge samples were directly exposed to ultraviolet radiation. The optimal irradiation duration was obtained through several experiments, and the influence of ultraviolet irradiation on the degree of cracking of the original sludge was studied.

### 2.4. Method of Index Determination

The following methods were used to measure the parameters and indicators in this study: the supernatant was filtered through a 0.45 mm microporous filter after centrifugation; the pH value was measured with a pH meter; Folin’s phenol reagent method [17] was used to determine the protein and humic acid content, with bovine serum protein and humic acid as the standard solutions; and polysaccharides were determined by the anthrone-sulfuric acid method with glucose as the standard solution. The moisture content of sludge was measured by the oven dry method [18]. All experiments were repeated three times under the same conditions, and the data reported in this study are the mean values of the three repeated measurements.

#### 2.4.1. Protein and Humic Acid Detection Method

The sCOD was measured by a multiparameter water quality tester, and the experiments were carried out using the reagents (LH-E and LH-D) supplied with the tester. A total of 2.5 mL of the water sample to be tested was added to a digestion tube, 4.8 mL of LH-E reagent was added, the tube was shaken manually and then it was put into the detector to preheat at 100 °C for 10 min to ensure that the residual hydrogen peroxide was removed. Then, 0.7 mL of LH-D reagent was added and shaken well, and the sample was eliminated for 10 min at 165 °C. The sample was allowed to cool down for 2 min, and then 2.5 mL of distilled water was added to the digestion tube. The tube was again shaken well and measured after cooling for 2 min.

#### 2.4.2. Protein and Humic Acid Detection Method

Protein and humic acid were determined by the same method and calculated by different formulas. Two sets of 10 mL centrifugal tubes were used. An amount of 2 mL of tester and 2.8 mL of Lowry reagent 1 were added into the set of tubes. An amount of 2 mL of tester and 2.8 of ml Lowry reagent 2 were added to the second set of tubes. After full mixing, they were left for 10 min at room temperature. A total of 2.0 mL of ultrapure water was used as a blank sample according to the same color rendering operation. Then, 0.4 mL of Folin’s phenol reagent was added into the two sets of centrifuge tubes. After dosing, they were left at room temperature for 30 min and readings were obtained at 750 nm. Group 1 was denoted as *A*_1_, group 2, as *A*_2_, the absorbance of the protein in the sample was denoted as *A_protein_*, and the absorbance of humic acid was denoted as *A_humic-acid_*:

$$\begin{array}{l} A_{protein}=1.25\times \left(A_{1}-A_{2}\right)\\ A_{humic-acid}=1.25\times A_{1}-0.25\times A_{2} \end{array}$$

#### 2.4.3. Polysaccharide Detection Method

Firstly, an anthrone reagent was prepared and 0.1 g of anthrone was added to 100 mL of concentrated sulfuric acid solution. Test drugs should be used on the spot and kept away from light. A total of 2.0 mL of tester of solution was taken and 6 mL of anthrone reagent was added into a 10 mL centrifuge tube for uniform shock, then removed after 15 min of boiling in a water bath and another 15 min in an ice water bath. The readings were detected at 625 nm. An amount of 1.0 mL of ultrapure water of the same color rendering operation was used as a blank sample.

### 2.5. 3D-EEM Fluorescence Spectroscopy

The 3D-EEM employed in this study used a 150 W ozone-free xenon lamp as the excitation light source. The parameters were set as the following: the excitation wavelength range (Ex) was from 200 nm to 600 nm, with a 3 nm interval, while the emission wavelength (Em) was fixed at 200–800 nm, with a 1.17 nm interval and a 5 nm slit width. The scanning speed was 1200 nm·min^−1^. Raman scattering and first- and second-order Rayleigh scattering were deducted by using ultrapure water as a blank sample, and the internal rate was corrected.

### 2.6. SEM Detection Method

The pretreated sludge mixture was centrifuged to extract organic matter, and the solid sludge was stored separately. Gradient dehydration was carried out on the sludge. The samples were immersed in 4% glutaraldehyde phosphate buffer and stored at 4 °C for 10–12 h for fixation. The samples were rinsed and centrifuged at 5000 rpm for 5 min, and the supernatant was poured off. The samples were rinsed with phosphoric acid buffer 3 times for 10 min each time. Gradient ethanol was used for dehydration. The rinsed samples were put into a gradient of 50%, 70%, 80% and 90% ethanol for dehydration once and finally 100% ethanol three times, for 10–15 min each time. Finally, the samples were vacuum dried at 65 °C for 12 h and sputtered for electron microscopy.

An EVO-18 scanning electron microscope (SEM) and a five-quadrant backscattered electron detector (BSE) were used to obtain the signals of secondary electrons and backscattered electrons from the samples. The appearance and morphology of the pretreated sludge were observed under the condition of amplification of 20,000 times. In the process of determination, the sample was sputtered to enhance the electrical conductivity. The electron microscope in this study was operated at acceleration voltages of 5 kV and 15 kV, with working distances of 9.3 mm and 9.4 mm, respectively.

### 2.7. Detection of Sludge Particle Size

The particle size of the sludge was analyzed using the Mastersizer 2000 (Malvern, UK) laser particle size analyzer. An appropriate amount of the solid sludge sample was taken and washed with ultrapure water three times to remove impurities, from which 2 mL of the rinsed solid sample was removed and 8 mL of 0.05 mol/L sodium hexametaphosphate solution was added as a dispersant. Measurements were carried out with the aid of an ultrasound, and the average value was taken from three separate measurements.

## 3. Discussion

### 3.1. The Optimal Experimental Conditions of Pure Fenton

EPS mainly play the role of a skeleton in sludge. Most microorganisms will gather in EPS to form a biopolymer [19], and the aggregates also contain substances absorbed by foreign sewage [2]. The main components of EPS include proteins, polysaccharides, humic acids and nucleic acids, of which proteins and polysaccharides account for 70–80%, and humic acids, about 20% [20]. Simultaneous pretreatment of microbial cells and EPS in the sludge floc structure by Fenton and its derivative method, UV-Fenton, fully solubilized the rich organic matter out of the liquid phase. When determining the experimental conditions for each of the Fenton reactions, unreacted H_2_O_2_ would remain in the sludge, a highly concentrated medium, and would interfere with the sCOD data [21], preventing accurate conclusions from being drawn. In this study, the residual H_2_O_2_ was removed by heating [22].

#### 3.1.1. The Dosage of Fe^2+^

With the pH of the sludge set at 3, the dosage of H_2_O_2_ at 7.5 g/L (the initial concentration of H_2_O_2_ in the sludge samples), the reaction time at 50 min and the temperature at 20 °C, Fe^2+^ dosages of 1.3 g/L, 1.4 g/L, 1.5 g/L, 1.6 g/L and 1.7 g/L were separately applied to investigate the influence of different concentrations of Fe^2+^ on the improvement in sludge performance by Fenton to ultimately determine the optimal concentration of Fe^2+^.

**Figure 2:**
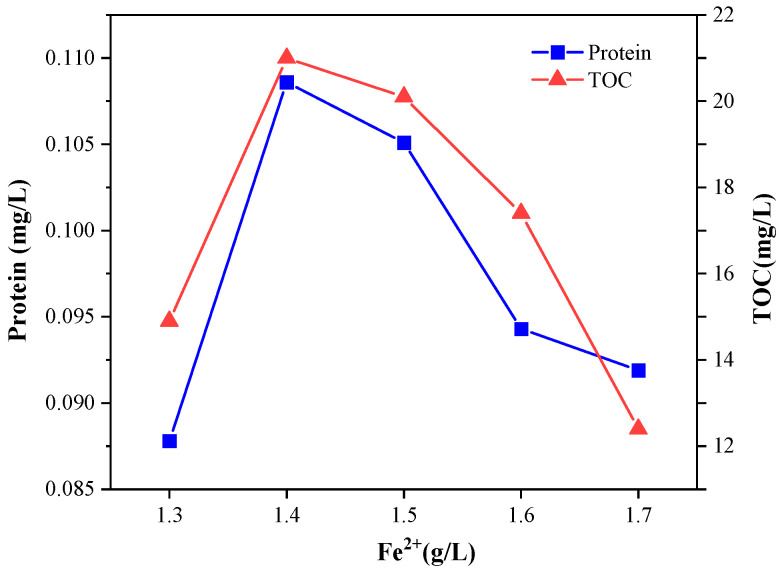
Effect of Fe^2+^ concentration on protein concentration and TOC in the sludge during the Fenton reaction.

It can be seen from Figure 2 that the optimal Fe^2+^ concentration for the Fenton reaction was 1.4g/L, under which condition protein concentration was the highest of 0.109 mg/L and TOC, of 21 mg/L. When the concentration was less than 1.4 g/L, the protein concentration and TOC content were relatively low. Due to the insufficient concentration of Fe^2+^, the low content of ·OH in the reaction system slowed down the reaction speed and the Fenton reaction was inhibited. When Fe^2+^ concentration increased from 1.3 g/L to 1.4 g/L, TOC content significantly increased by 40.9%. When the Fe^2+^ concentration increased from 1.4 g/L to 1.7 g/L, the protein concentration decreased in turn. When the dosage of H_2_O_2_ remained unchanged and the concentration of Fe^2+^ gradually increased, the cracking of sludge with the Fenton process was mainly achieved through the gradual increase in strong oxidizing ·OH. However, when the concentration of Fe^2+^ was too high, it was oxidized into Fe^3+^ while reducing H_2_O_2_, weakening the oxidation capacity of the whole reaction system, reducing the cracking effect of the Fenton reaction and the protein concentration.

#### 3.1.2. The Dosage of H_2_O_2_

When the Fe^2+^ dosage was 1.4 g/L (optimal concentration), pH was 3, reaction time was 50 min and temperature was 20 °C, H_2_O_2_ solutions with concentrations of 4.5 g/L, 6 g/L, 7.5 g/L, 9 g/L and 10.5 g/L were added, respectively. The influence of H_2_O_2_ dosage on Fenton’s sludge cracking capacity was investigated, and the optimal dosage of H_2_O_2_ was determined. 

**Figure 3:**
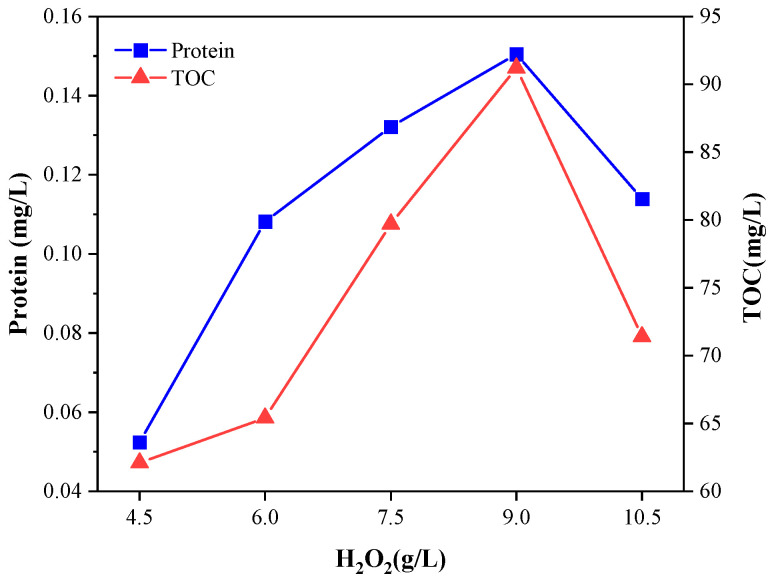
Effect of H_2_O_2_ dosage on protein concentration and TOC in the sludge during the Fenton reaction.

It can be seen from Figure 3 that the best dosage of H_2_O_2_ in the Fenton reaction was 9 g/L. When the dosage of H_2_O_2_ increased from 4.5 g/L to 9 g/L, the protein concentration and TOC content increased successively. When the dosage was 9 g/L, the highest protein concentration was 0.151 mg/L, the TOC was 91.2 mg/L, and Fenton had the strongest cracking effect. When the dosage of H_2_O_2_ increased from 9 g/L to 10.5 g/L, the protein concentration decreased to 0.114 mg/L, with a decrease of 32.4%. Excessive H_2_O_2_ in the reaction system oxidized Fe^2+^ into Fe^3+^, which rapidly increased the concentration of Fe^3+^ and reduced the probability of the catalytic reaction between H_2_O_2_ and Fe^2+^, reducing the production of ·OH and weakening the oxidation capacity of Fenton. Meanwhile, the excess H_2_O_2_captured ·OH and generated O_2_, reducing the utilization rate of H_2_O_2_ [23] and the cracking ability of the Fenton reaction on sludge.

#### 3.1.3. The Reaction Times

Under the reaction conditions of an Fe^2+^ dosage of 1.4 g/L, an H_2_O_2_ dosage of 9 g/L, a reaction pH of 3 and a temperature of 20 °C, the reaction times of the sludge treatment with Fenton were set at 30, 35, 40, 45, 50 and 55 min, respectively, to study the effect of reaction time on Fenton’s ability to crack sludge. 

**Figure 4:**
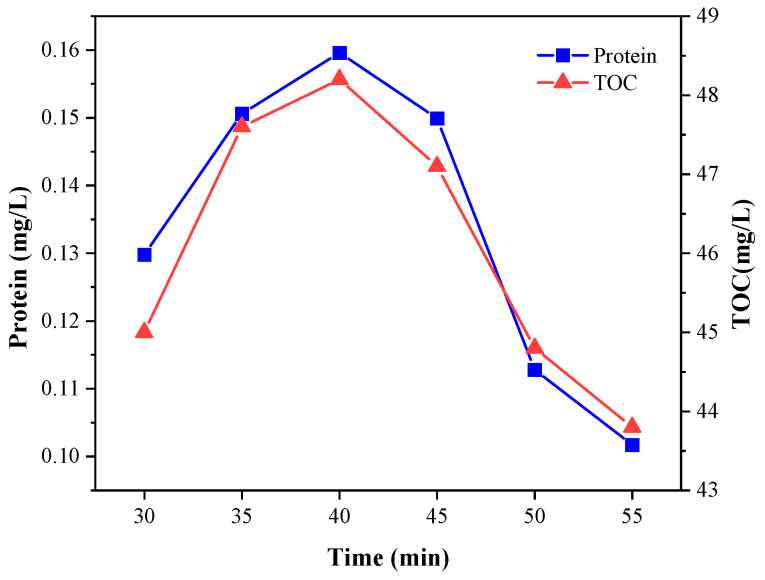
Effect of the Fenton reaction time on protein concentration and TOC in sludge.

It can be seen from Figure 4 that when the reaction time increased from 30 to 40 min, the protein concentration and TOC gradually increased, and the cracking effect was the best at 40 min when the protein concentration was 0.16 mg/L and the TOC was 48.2 mg/L. When the reaction time was between 40 and 55 min, the protein concentration and TOC decreased rapidly. It can be inferred that in the initial process of the Fenton reaction, the content of ·OH produced by H_2_O_2_ was abundant. Therefore, the reaction speed was faster, the sludge cracking degree was stronger, and the protein concentration and TOC content increased between 30 and 40 min. With the passage of reaction time, ·OH was gradually consumed, Fenton’s cracking of the sludge was gradually slowed down and part of the protein in the solution was oxidized, reducing the concentration.

### 3.2. The Optimal Reaction Time of UV-Fenton

On the basis of the optimal reaction conditions of the Fenton method derived from the above experiments, the effect of reaction duration on the sludge’s cracking ability of UV-Fenton was explored under the irradiation of a UV lamp with a power of 10 W.

**Figure 5:**
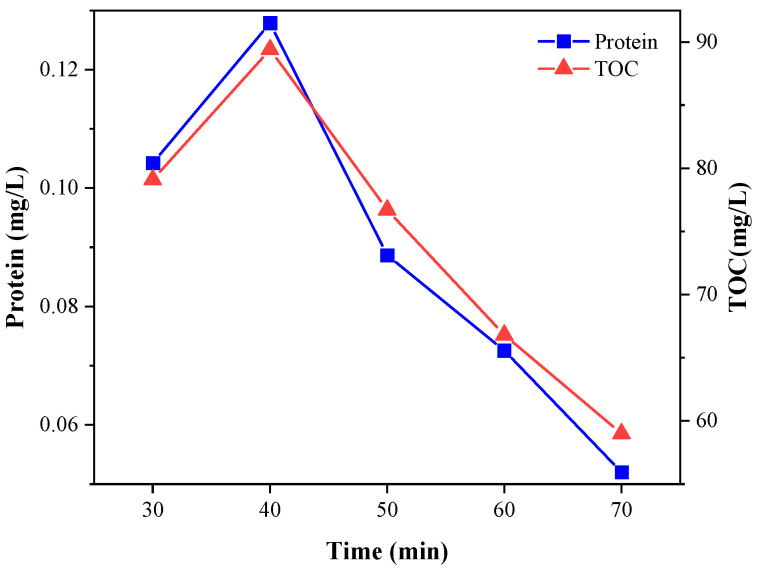
Effect of UV-Fenton reaction time on protein concentration and TOC in sludge.

As can be seen from Figure 5, UV-Fenton was most effective in leaching organic matter from the sludge under UV lamp irradiation at a reaction time of 40 min and did not destroy the organic matter itself, at which time the protein concentration was 0.13 mg/L and the TOC concentration was 89.4 mg/L. As the oxidation reaction of UV-Fenton continued, the concentrations of sCOD and TOC decreased with time, and the decrease in protein concentration (43.2%) was greater than that of Fenton (29.3%) in 40–50 min, suggesting that the oxidative capacity of Fenton for organic matter was enhanced by ·OH with the assistance of the UV lamp.

### 3.3. Basic Component Analysis

#### 3.3.1. Protein, Humic Acid and Polysaccharide

**Figure 6:**
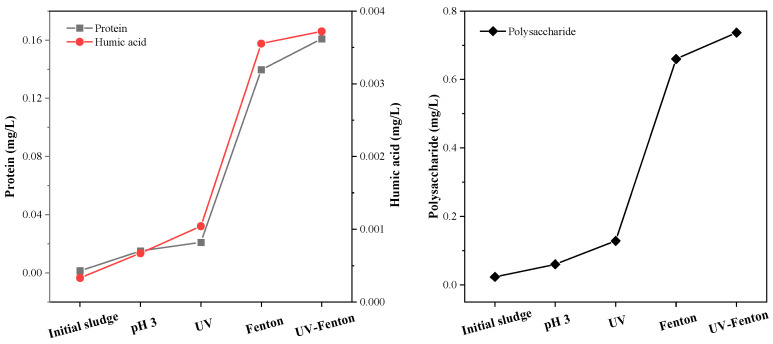
The content of protein, humic acid and polysaccharide in sludge following different pretreatment methods. Initial sludge is sludge that has not been pretreated.

The content of proteins, polysaccharides and humic acids was analyzed to determine the degree of dissolution of organic matter in the sludge by different pretreatment methods. In the experiment, the optimal acidity of the Fenton process was 3. A separate experimental group with a pH of 3 and UV light was set up to judge the degree of influence of the acidic reaction environment and UV light on the experimental results during the Fenton reaction process. It can be seen from Figure 6 that the Fenton group and the UV-Fenton group had the most protein, polysaccharide and humic acid content produced from sludge cracking after pretreatment. The experimental group with the best effect was the UV-Fenton group, in which the concentrations of the three components were 0.16 mg/L, 0.74 mg/L and 3.7 × 10^−3^ mg/L, respectively. Although UV-Fenton could substantially leach the organic components from sludge into the liquid phase, it was similar to the Fenton protein content of 0.14 mg/L. This was followed by single UV and finally the experimental group with a pH of 3. The trends of humic acids and polysaccharides were consistent with those of proteins, suggesting that the acidic environment and single UV light had little effect on the sludge and that it was the Fenton reaction that was at work. In the four experimental groups, the best UV-Fenton had 110 times more protein, 32 times more polysaccharide and 12 times more humic acid than the original sludge sample. Within the optimal reaction time of 40 min, Fenton and UV-Fenton had the best effects on cell wall cracking. In the appropriate time, the released proteins were not further oxidized and decomposed into small molecules, and the dissolved organic matter was retained to the greatest extent [24]. Figure 6 shows that UV had an assisting effect on Fenton, but the effect was not strong in terms of protein content, which was only about 1.1 times that of Fenton alone.

#### 3.3.2. sCOD and TOC

sCOD is an important index of sludge pretreatment, and the cracking degree of sludge pretreatment will directly affect sCOD. The microorganisms in sludge undergo lysis, during which the microbial cell contents are released, and the mixture produced by the sludge lysis contains rich sCOD [25].

**Figure 7:**
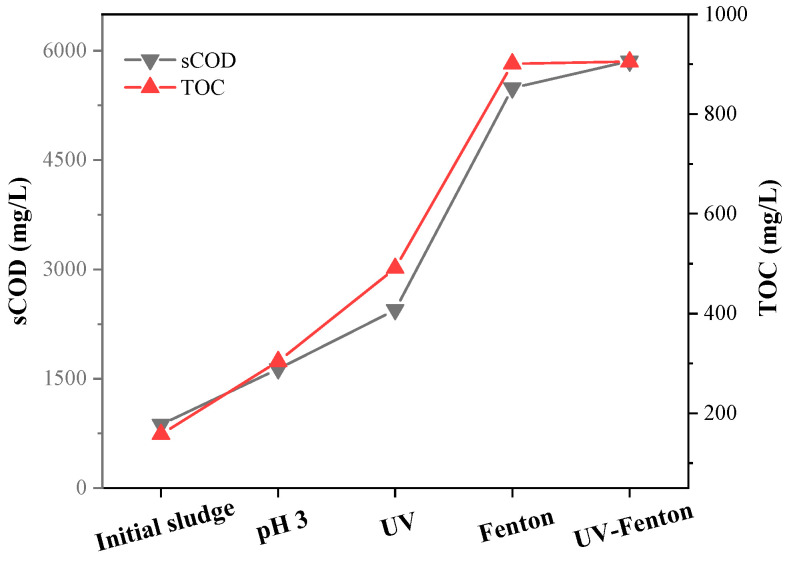
sCOD and TOC content in sludge following different pretreatment methods.

Figure 7 shows the effects of different pretreatment methods on the sCOD and TOC of the sludge. The most obvious breaking effect was achieved by UV-Fenton, with the sCOD reaching 5856 mg/L, about seven times higher than the original sludge sample. The second best effect was Fenton, with the sCOD being six times higher than the original. Acid treatment was the least effective, with a sCOD of only about 1600 mg/L. Although UV had an effect on the Fenton reaction to crack sludge cells, there was no obvious improvement. UV-Fenton had the best solution-increasing effect on the sCOD, with the highest solubility. The TOC results show that the Fenton reaction was not affected by UV irradiation, and the content of the Fenton reaction was about 900 mg/L.

### 3.4. 3D-EEM Analysis

3D-EEM can characterize the location of fluorescence peaks of most dissolved organic compounds in the natural environment, such as aromatic proteins [26] and humic acids [27]. The regional division of fluorescence peaks is shown in Table 1.

3D-EEM can be used for quantitative analysis, and the maximum fluorescence intensity, fluorescence peak location and intensity of different peaks can be obtained from spectral diagrams [28]. In this study, each 3D-EEM gave spectral information on the chemical composition of the sludge samples.

**Figure 8:**
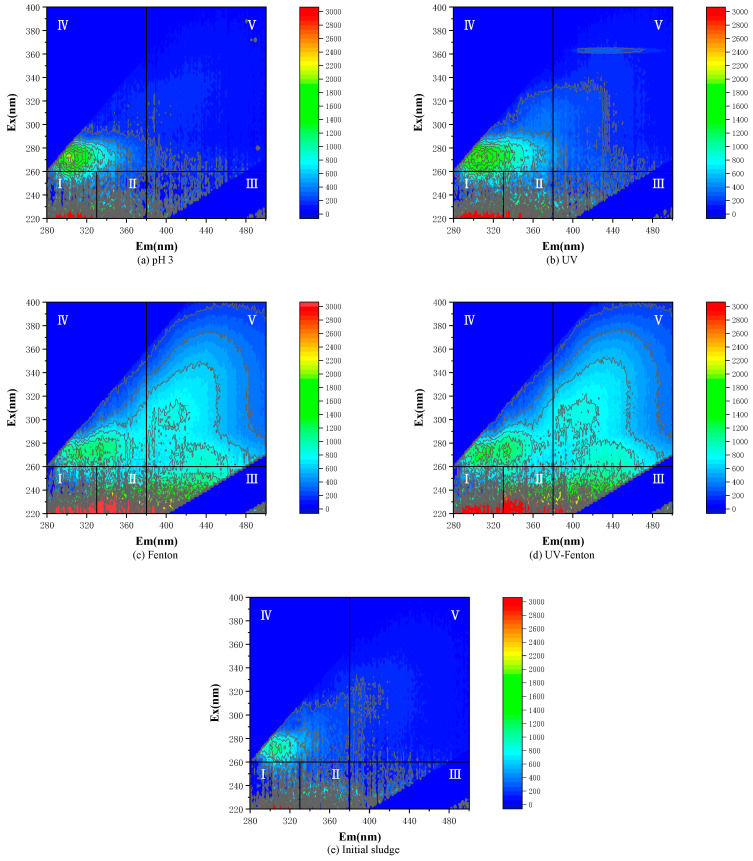
3D-EEM of sludge after different pretreatment methods.

As shown in Figure 8e, the fluorescence intensities in regions I, II and IV are relatively obvious, and the maximum fluorescence peak appears in region I, indicating that the components of the untreated sludge liquid phase components were mainly aromatic proteins (A and B), followed by various dissolved microbial metabolites (D). As shown in Figure 8a–d, the fluorescence peaks in each region were enhanced to varying degrees, among which the content of proteins all increased.

In Figure 8c,d, the biggest change is in region V, where the fluorescence intensity response was greatly improved and the peak value was larger. The concentration of humic acid in the untreated sludge was low, and the fluorescence intensity was weak. After Fenton and UV-Fenton treatment, the peak value of region V is gradually obvious, indicating an increase in the concentration of humic acid. However, compared with Figure 8a,b, the peak value in region IV decreases and the fluorescence intensity became weaker, indicating that the microbial cracking effect in the sludge after Fenton oxidation reaction was the most effective and the content of dissolved microbial metabolites decreased. A comparison of the spectrograms of the four treatments and the original sludge samples shows that the UV-Fenton method had a substantial increase in all fluorescence intensities in regions I, II, III and V, but is similar to the spectrogram of the Fenton method. The 3D-EEM results reflecting changes in each component is consistent with the results of each assay.

### 3.5. Characterization by Electron Microscope

A field-emission scanning electron microscope (SEM) was employed to study the morphology of the activated sludge after the organic component extraction by the four treatment methods. Pretreatment of sludge is mainly aimed at achieving the cracking of the cellular structure and allowing the outflow of organic components which can be utilized [29]. 

**Figure 9:**
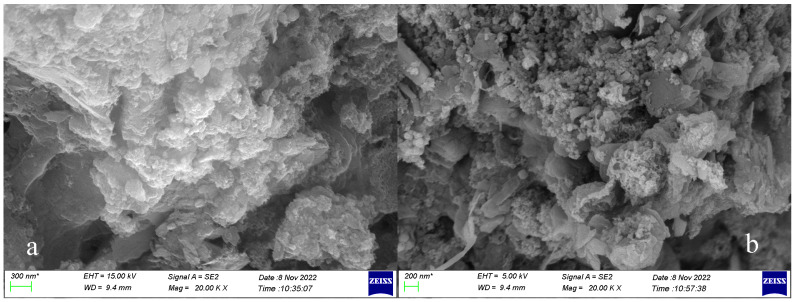

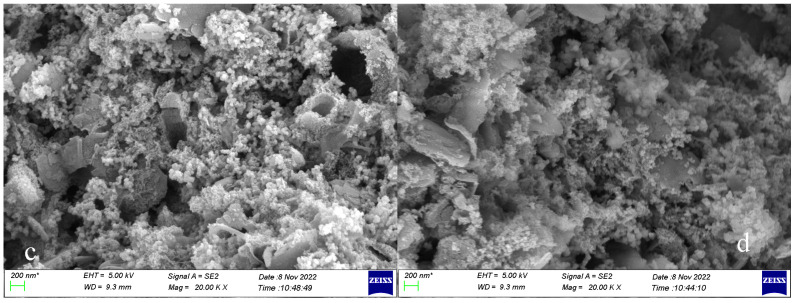
Sludge field-emission scanning electron microscope analysis after different pretreatments: (**a**) pH 3, (**b**) UV, (**c**) Fenton, (**d**) UV-Fenton.

In Figure 9, it can be clearly seen that the surface of the sludge was cracked at different degrees. The SEM images of the sludge treated by various pretreatment methods and the original sludge show that the surface shape of the original sludge was smooth, the porosity was small and the results were compact, indicating that the shape of the sludge cells was relatively complete. Figure 9a,b shows the cracking degree of the sludge under acidic and ultraviolet irradiation conditions. The surface of the sludge was cracked to a certain extent and the porosity was increased, but the coverage rate of the cracked sludge was low. However, in Figure 9c,d, the sludge was treated by Fenton and UV-Fenton, showing a great degree of breakage. The appearance of the sludge was no longer smooth, and the sludge cells were broken into a flocculent shape. This indicates that the structure of the sludge cells was destroyed to a great extent. On the other hand, advanced oxidation was effective in cracking the sludge.

### 3.6. Sludge Particle Size and Water Content after Pretreatment

A laser particle size analyzer produces a different angular distribution of scattered light through different particle sizes in the beam, with large particles scattering light at a small angle and small particles scattering light at a large angle [30]. A series of photodetectors are installed at different angles to receive the scattered light at different angles and the scattered light intensity can bemathematically inverted to obtain the particle size distribution [31].

**Figure 10:**
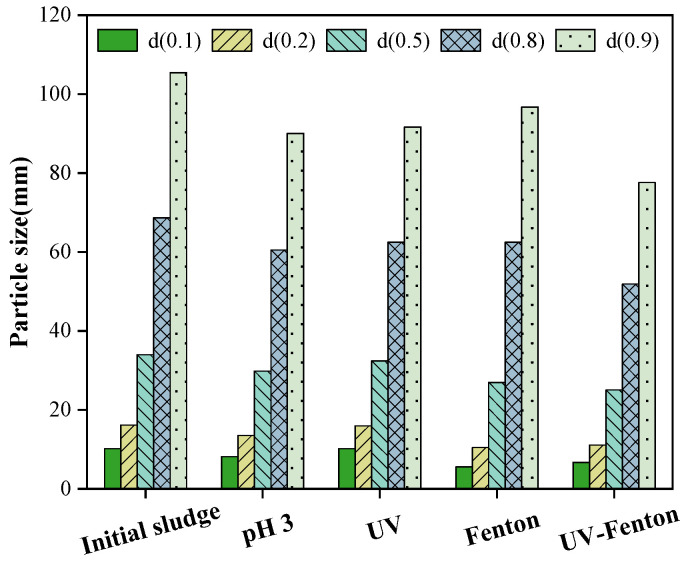
Trends of laser particle size changes.

The results are shown in Figure 10. Compared to the original sludge, the single pH of 3 and single UV light groups did not change significantly in most volume occupancies, but the reduction in particle size was greater at 90% volume occupancy. This indicates that these two factors could only crack larger volumes of sludge and had a limited ability to crack sludge. The two pretreatment methods, Fenton and UV-Fenton, affected the sludge particle size to a greater degree, with the degree of change being significant for most volume occupancies. Most notably, the decrease in particle size was most pronounced for volume occupancies in the range of less than 50%. When comparing the original sludge to the pretreated Fenton state at a 10% volume fraction, the sludge particle size decreased from 10.166 mm to 5.604 mm. The pretreated UV-Fenton sludge particle size cracked sludge particles better than the Fenton method over arange of 20–90% of the volume fraction.

**Figure 11:**
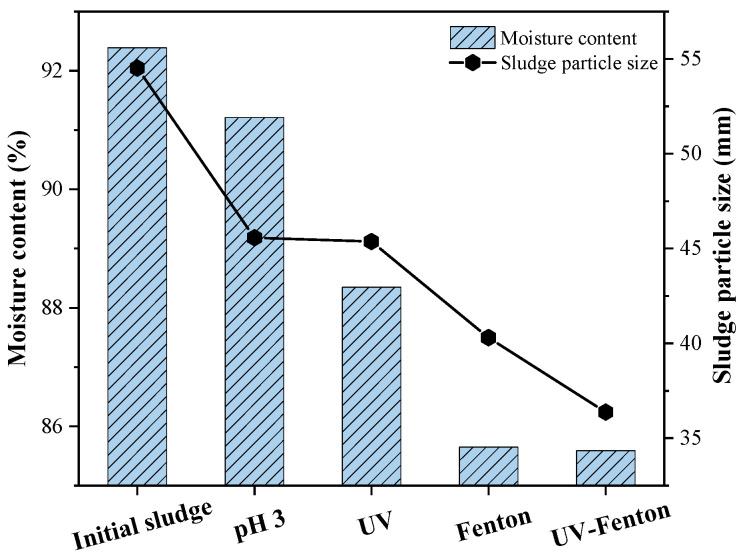
Comparative plots of sludge water content and average particle size.

As shown in Figure 11, the pretreatment method with the best cracking effect from the point of view of the average sludge particle size was UV-Fenton, which was more capable of cracking the sludge cells than the single Fenton method, and the average particle size was reduced from 54.52 mm to 36.37 mm. The effects of Fenton and UV-Fenton on sludge dewatering were similar when analyzed from the perspective of sludge water content. The decrease in sludge water content after pretreatment indicates that Fenton and UV-Fenton caused the sludge floc structure to lose its original water-locking property, resulting in enhanced sludge dewatering. Analyses from these two perspectives indicate that Fenton and UV-Fenton are very effective in achieving sludge reduction.

## 4. Conclusions

This study describes how effective the UV-Fenton and Fenton processes are in cracking activated sludge. The experimental results showed that the most effective treatment method was the UV-Fenton method, and the optimal Fenton cracking conditions were the following: 1.4 g/L of Fe^2+^ and 9 g/L H_2_O_2_ were added at a pH of 3, and the reaction was carried out for 40 min, which resulted in a protein concentration of 0.66 mg/L and an sCOD of 5489 mg/L. In the case of the synergistic effect of UV under the condition of UV synergism, the pretreatment protein concentration was 0.74 mg/L and the sCOD was 5856 mg/L, which was 15.2%, 11.7%, 2.8% and 6.7% of the single Fenton method. In the 3D-EEM fluorescence detection analysis, it was found that the fluorescence intensity of sludge that was pretreated by Fenton and UV-Fenton increased significantly in regions I and II and peaked in region V, and the fluorescence intensity was gradually increased, which more intuitively reflects that the sludge treated by single Fenton and UV-Fenton had a positive effect on the disintegration of the organic matter such as proteins and humic acid, etc., which proves that three-dimensional fluorescence spectroscopy can be used for the analysis of sludge. This demonstrates that 3D-EEM is a suitable and effective method for characterizing the complex composition of sludge. The sludge particle size was reduced from the original 54.52 mm to 40.30 mm and 36.37 mm, respectively.

## Figures and Tables

**Table 1:** Fluorescence peak region divisions.

Area	Range Ex/Em (nm)	Fluorescent Substance
I	220–250/280–330	Aromatic protein I (A)
II	220–250/330–380	Aromatic protein II (B)
III	220–250/380–500	Quasi-fulvic acid (C)
IV	250–280/280–380	Dissolved microbial metabolites (D)
V	250–400/380–500	Humic acid (E)

## Data Availability

Data are contained within the article.
